# Supplementary material for: The genome of cowpea (Vigna unguiculata [L.] Walp.)
Source: Plant J. 2019 May 28;98(5):767–82. doi: 10.1111/tpj.14349 (PMC6852540; doi:10.1111/tpj.14349)
Supplement: Supplementary file 1 — Figure S1. Estimation of cowpea genome size using flow cytometry. Figure S2. 27‐mer distribution of occurrences. Figure S3. Pre‐filter PacBio read length distribution. Figure S4. Post‐filter PacBio read length distribution. Figure S5. Subread Filtering PacBio read length distribution. Figure S6. Post‐filter PacBio read quality distribution. Figure S7. Synteny view between cowpea and common bean using the previous chromosome nomenclature. Figure S8. Gene and repeat densities, and recombination rate in the cowpea genome. Figure S9. SNP distribution in the cowpea genome. Figure S10. Cowpea pseudochromosome Vu03 reconstructed from 10 genetic maps using ALLMAPS. Figure S11. PCR amplification of the regions surrounding the two breakpoints of the inversion. Figure S12. Comparison between cowpea and adzuki bean for the cowpea inversion region. [file TPJ-98-767-s001.docx]

**Figure S1.**  **Estimation of cowpea genome size using flow cytometry**. Histogram obtained after flow cytometric analysis of prodium-iodide stained suspensions of cell nuclei isolated from *V. unguiculata* and *S. lycopersicum*. While peaks representing nuclei in G1 phase of cell cycle are clearly visible, peaks representing nuclei in G2 phase are small, indicating the presence of only a small fraction of cycling cells and/or cells arrested in G2 phase in leaf tissues. Average G1 peak positions were 100.3 and 150.1 for *V. unguiculata* and *S. lycopersicum*, respectively.

Relative DNA content (PI fluorescence)

Number of events


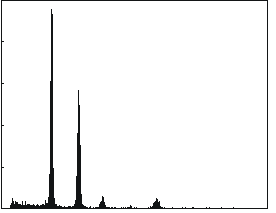


*V. unguiculata* (G1)

*S. lycopersicum* (G1)

*V. unguiculata* (G2)

*S. lycopersicum* (G2)

80

160

240

320

400

0

0

100

200

300

400

500

**Figure S2. 27-mer distribution of occurrences.** Distribution of occurrences of 27-mers in 168 M 149-bp paired-end reads produced with KAT (<https://github.com/TGAC/KAT>). The total number of base pairs is about 50 x10^9^. The x-axis represents the 27-mer multiplicity, the y-axis represents the number of 27-mers with that multiplicity. The peak of the distribution is 56, which represents the effective coverage. The total number of 27-mers in the range x=2-10000 is 31.381 x 10^9^ bp (27-mers that appear only once are considered erronous, i.e., to contain sequencing errors). The estimated genome size is thus 31.381 x 10^9^/56 = 560,379,733 bp.

**Figure S3. Pre-filter PacBio read length distribution.**

**
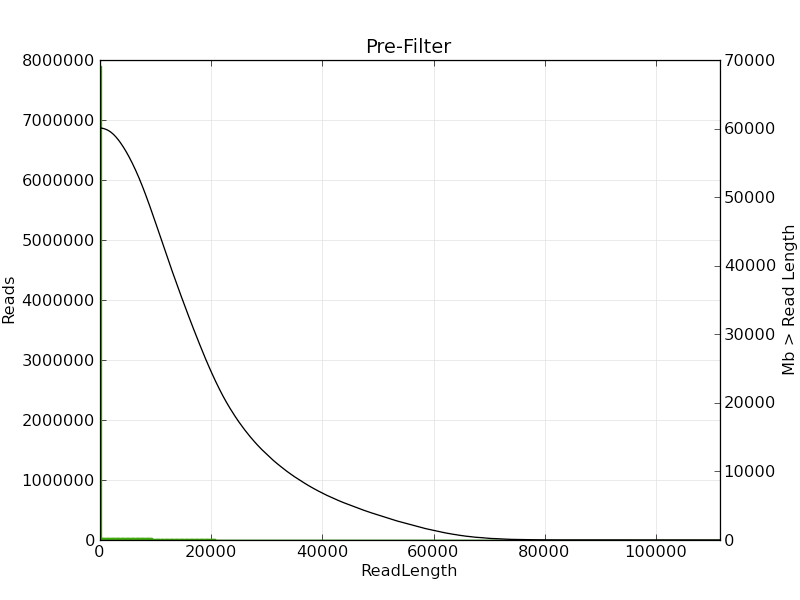
**

**Figure S4.**  **Post-filter PacBio read length distribution**.

**
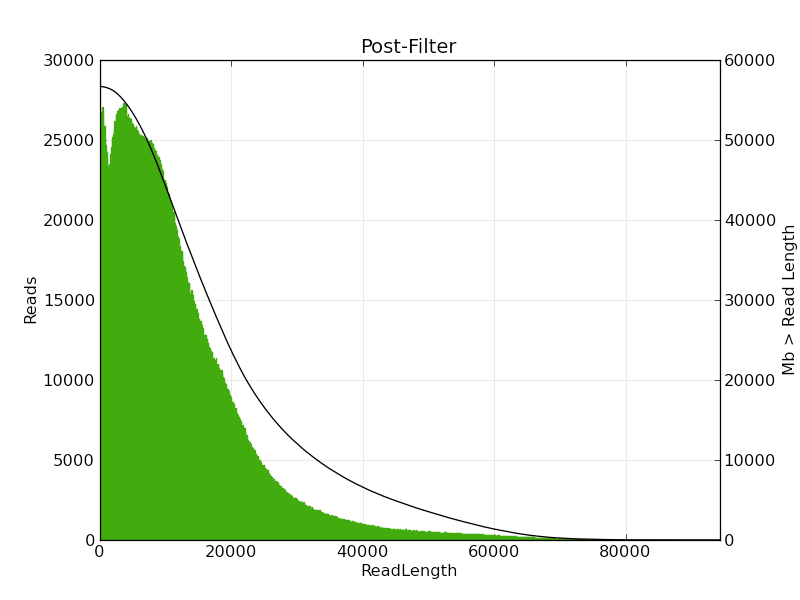
**

**Figure S5. Subread Filtering PacBio read length distribution.**

**
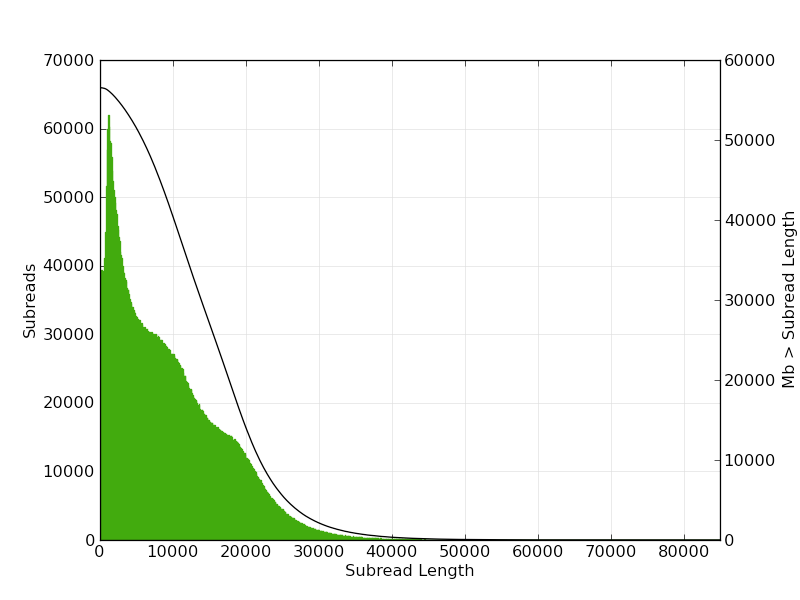
**

**Figure S6. Post-filter PacBio read quality distribution**.

**
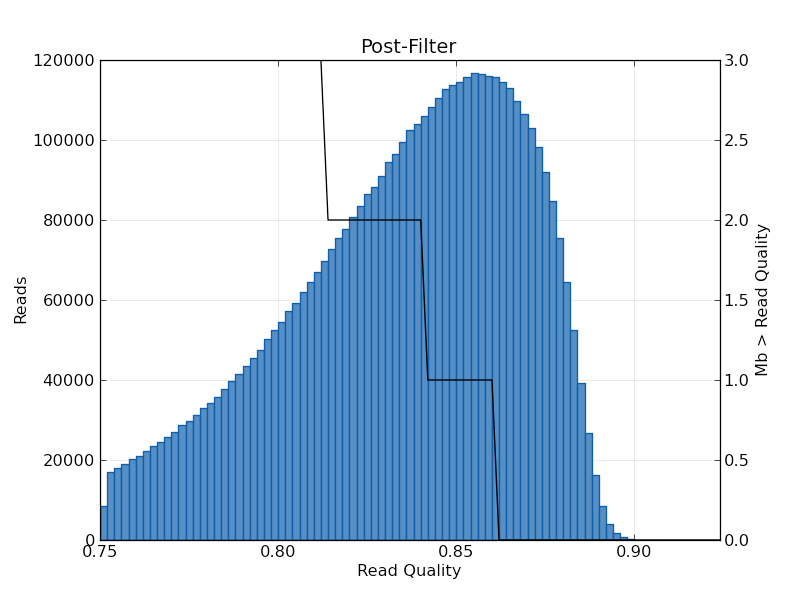
**

**Figure S7.** **Synteny view between cowpea and common bean using the previous chromosome nomenclature.** Synteny view between cowpea (Vu) and common bean (Pv) chromosomes using the previous cowpea chromosome numbering of Muchero et al. (2009) and Muñoz-Amatriaín et al. (2017). (A) Circos illustration of synteny. (B) Cowpea chromosomes painted based on syntenic relationships with common bean chromosomes (in different colors). Only syntenic regions with a exact match of 100 bp and a minimum alignment >1 kb are colored. Chromosomes indicated with arrows were inverted to meet the convention “short arm on top” based on the BAC-FISH analysis of Iwata-Otsubo et al. (2016).

**
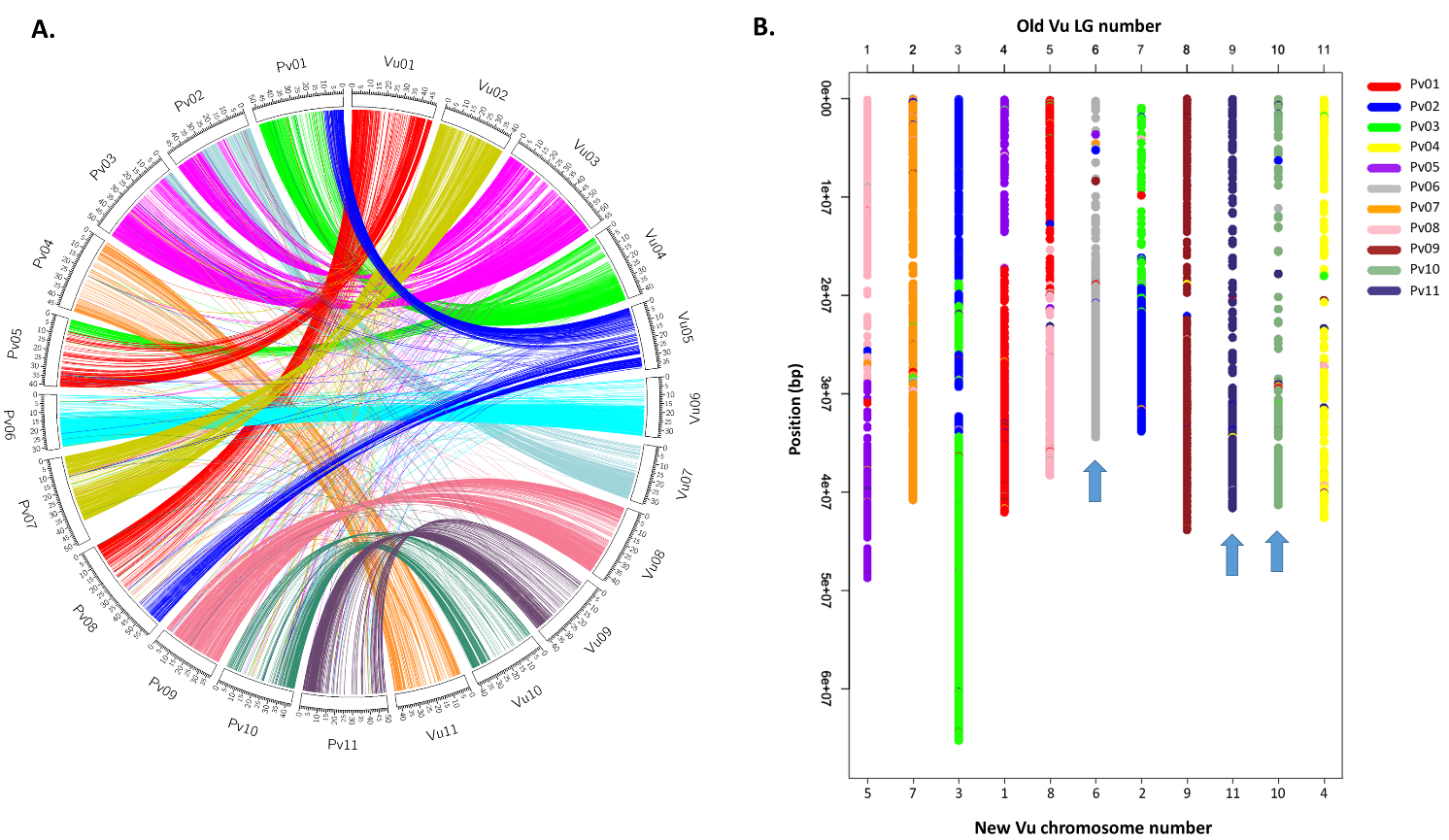
**

**Figure S8.** **Gene and repeat densities, and recombination rate in the cowpea genome.** Comparison between gene density (green line), repeat density (blue line) and recombination rate (red line) across the 11 cowpea chromosomes. Gene and repeat density are measured in 1 Mb non-overlapping windows, while recombination rate is measured in non-overlapping windows of 100 kb using values from Data S1. Vertical lines delimit the predicted centromeric regions.

**
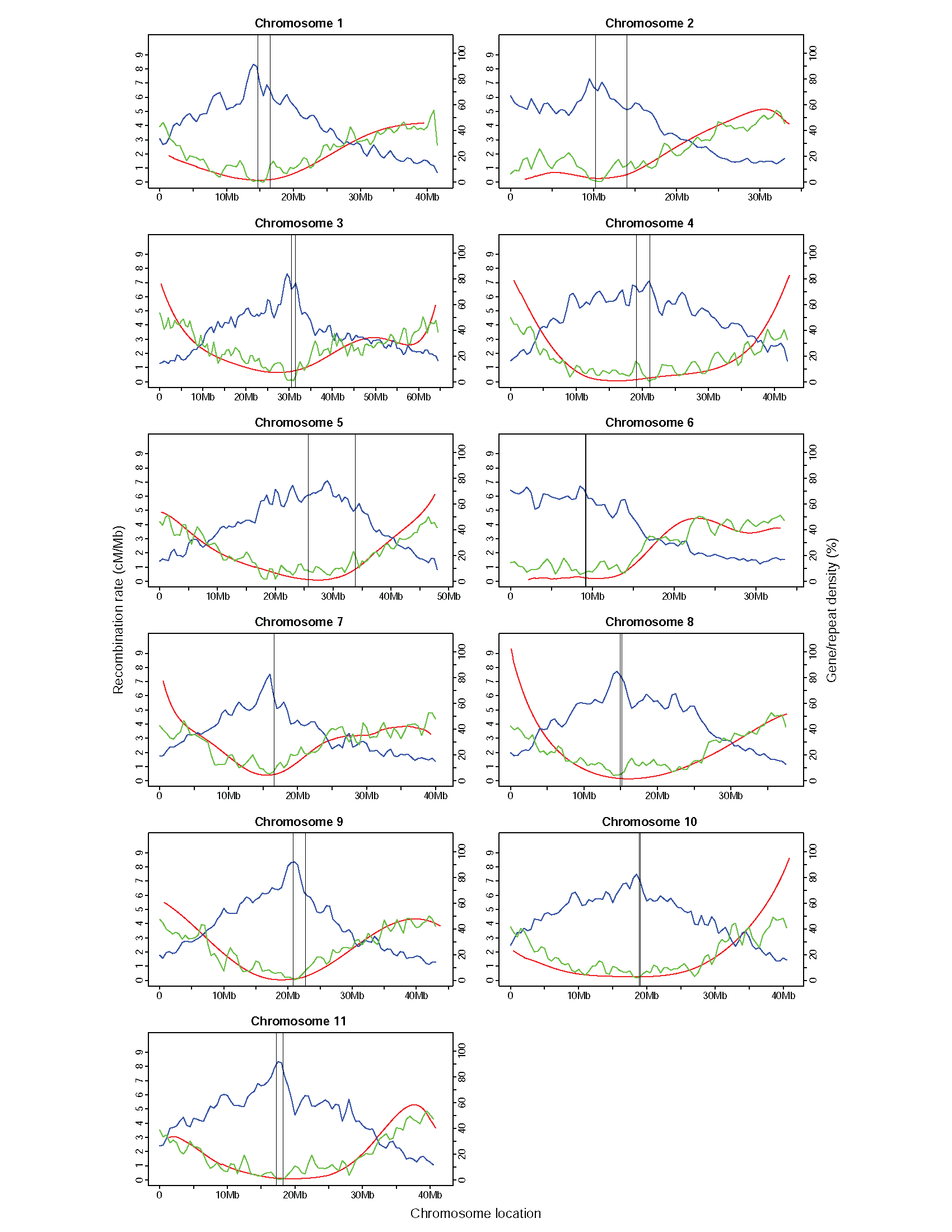
**

**Figure S9. SNP distribution in the cowpea genome.** Chromosome location of SNPs from the “1M list” (in red) and the Illumina iSelect Consortium Array (in blue). Arrows delimit the predicted centromeric regions.

**
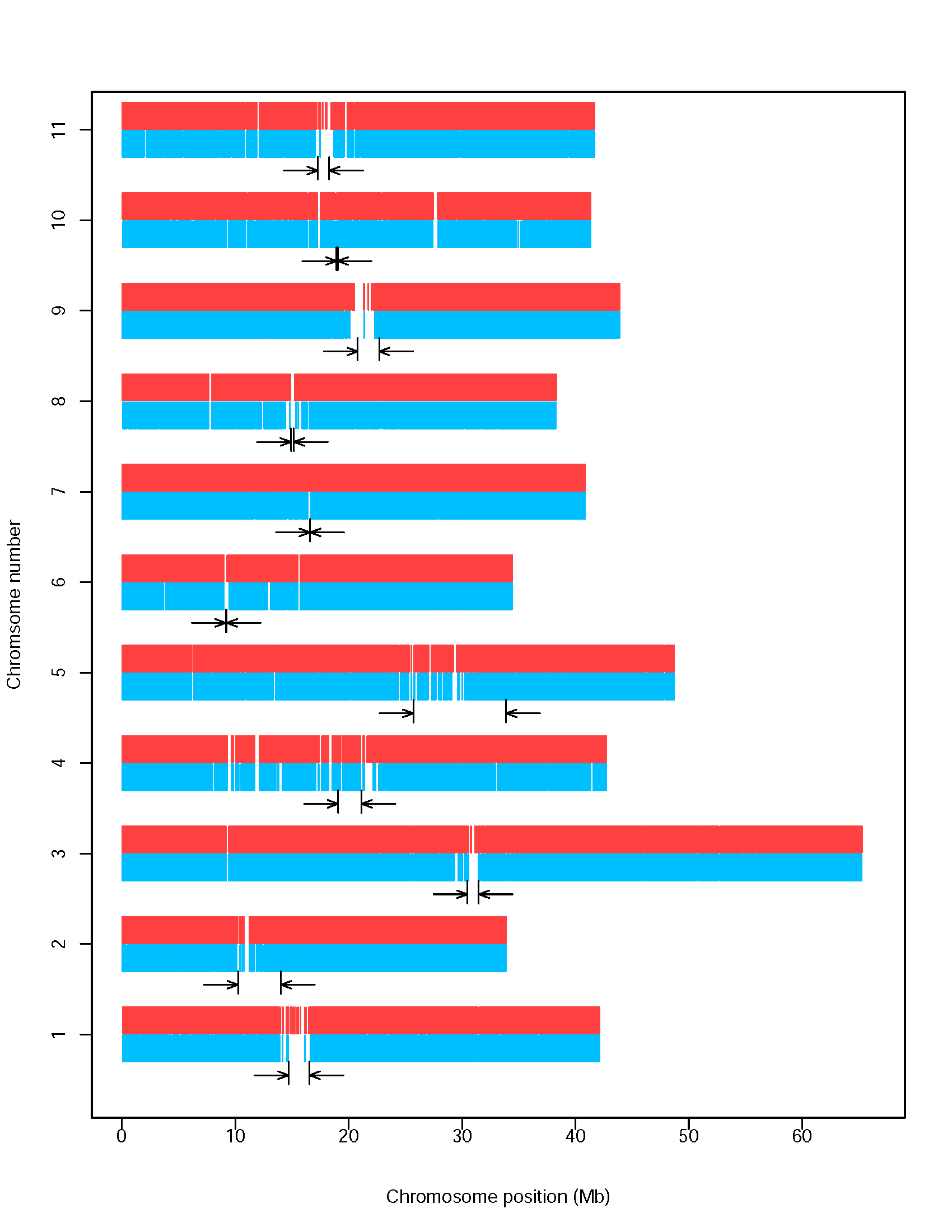
**

**Figure S10. Cowpea pseudochromosome Vu03 reconstructed from 10 genetic maps using ALLMAPS.** (A) Alignments between physical positions on pseudochromosome Vu03 and genetic map positions. (B) Scatter plots of genetic (y-axes) vs. physical (x-axes) map positions. Rho (*ρ*) represents the Pearson correlation coefficient. The chromosomal inversion can be observed in 7 out of the 10 genetic maps (red arrow). 27B=CB27xIT82E-18; 27I=CB27xIT97K-566-6; 27U=CB27xUCR779; 46I=CB46xIT93K-503-1; 5I=524BxIT84S-2049; NF=NullxFN-2-9-04; SV=SanzixVita7; TI=Tvu-14676xIT84S-2246-4; WC=IT99K-573-1-1xTVNu-1158; ZZ=ZN016xZhijiang282.

**
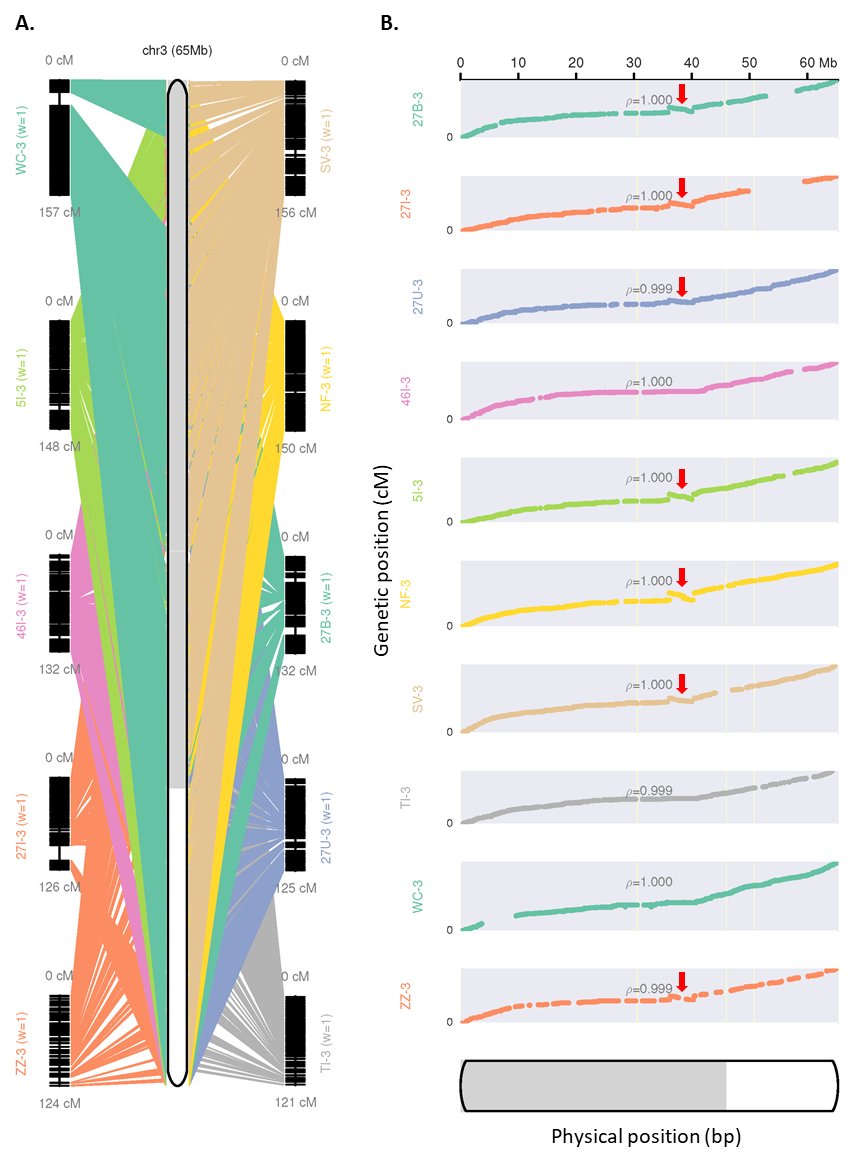
**

**Figure S11.** **PCR amplification of the regions surrounding the two breakpoints of the inversion.** Four accessions for each of the two orientations were tested. Type A refers to accessions having the same orientation as the reference genome, while type B indicates accessions having the opposite-to-reference orientation.

**
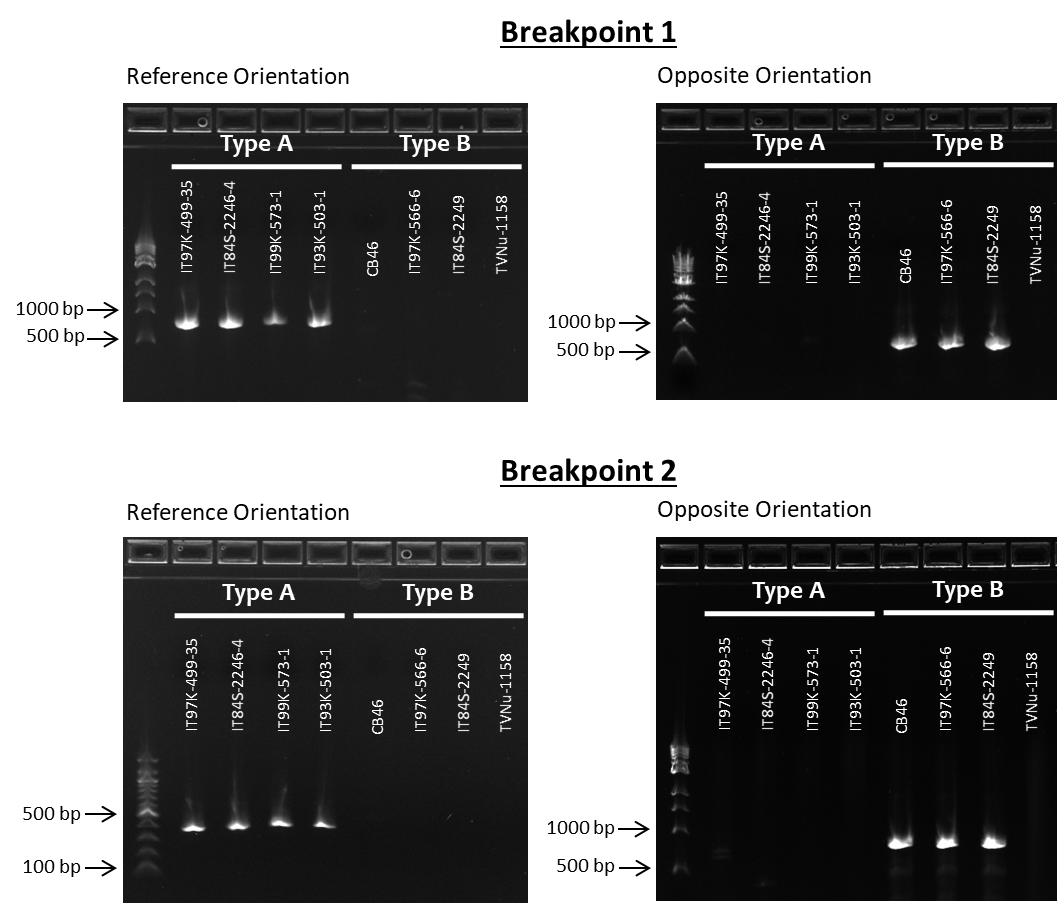
**

**Figure S12. Comparison between cowpea and adzuki bean for the cowpea inversion region.** Comparison between the Vu03 chromosomal inversion region in cowpea (y-axis) and its syntenic region in *Vigna angularis* (vigan.Shumari). Dots in the plot represent gene pairs likely to be orthologous between the two species, as assessed by their assignment  to the same gene family (represented by the dot colors) and their presence in these largely co-linear blocks. The break in the middle of the inverted segment (~36-41 Mbp in the *V. angularis* chromosome), corresponds to a block further translocated with respect to the cowpea region.

**
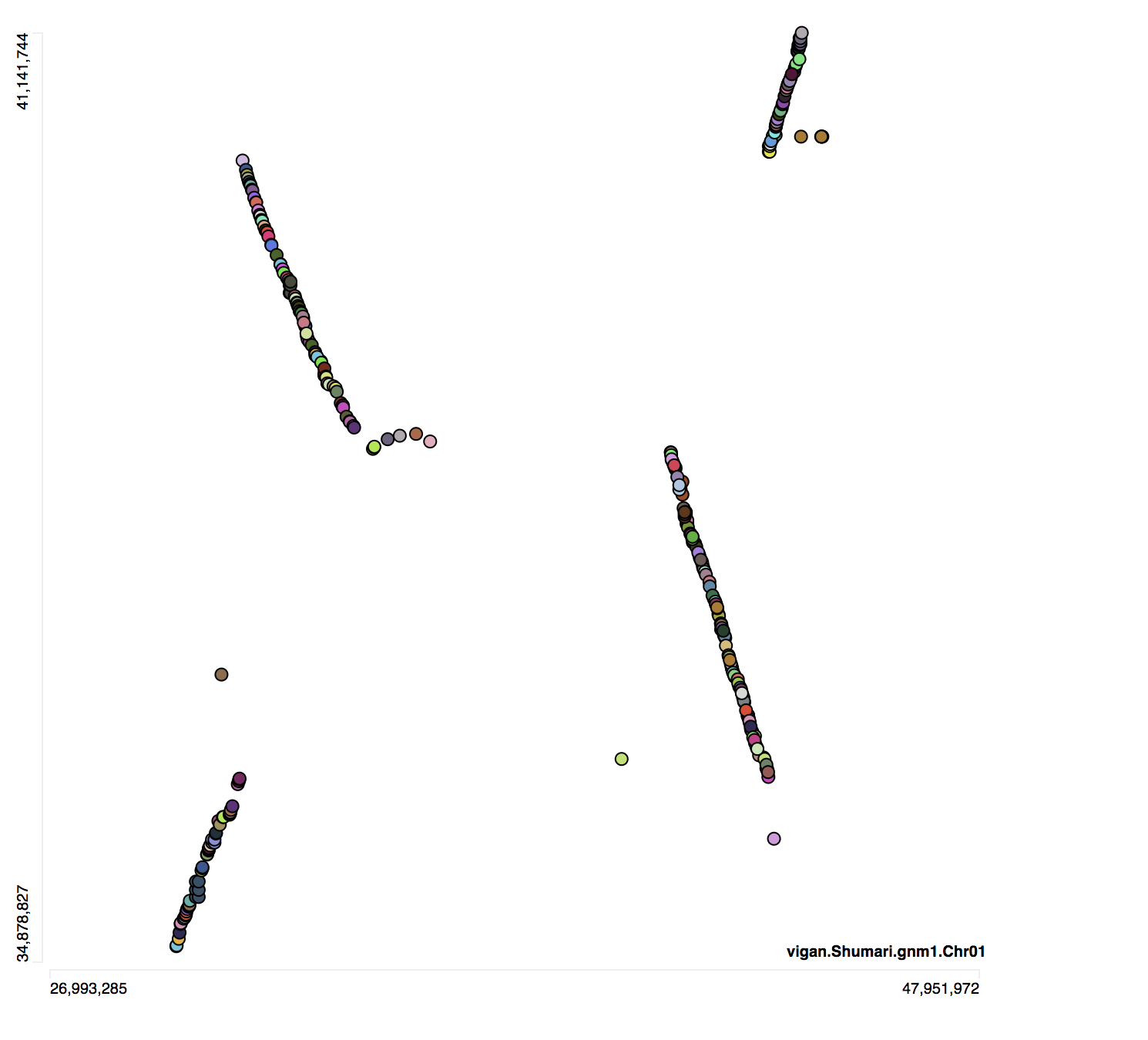
**
