# Supplementary material for: The genome of cowpea (Vigna unguiculata [L.] Walp.)
Source: Plant J. 2019 May 28;98(5):767–82. doi: 10.1111/tpj.14349 (PMC6852540; doi:10.1111/tpj.14349)
Supplement: Supplementary file 2 — Table S1. Statistics for BspQI optical map. Table S2. Statistics for the BssSI optical map. Table S3. Assembly statistics for the eight individual draft assemblies. Table S4. Characteristics of the 10 genetic maps used for pseudochromosome construction. Table S5. Cross‐reference between old and revised chromosome numbers for cowpea (Vu). Table S6. Annotated repeat abundances in cowpea. Table S7. Centromere position prediction. Table S8. Number and location of SNPs relative to annotated cowpea genes. Table S9. Comparative repeat abundance in Vigna species. Table S10. Primer sequences used to validate the Vu03 inversion. Table S11. Data sources and references for genome assemblies and annotations used in the gene family analysis. [file TPJ-98-767-s002.docx]

**Table S1.**  **Statistics for *BspQI* optical map.**

| # Molecules | 230,645 |
| --- | --- |
| Molecule N50 | 340.5 Kb |
| Molecule minimum length | 180 Kb |
| Molecule total length | 78.5 Gb |
| # BNG (optical map) contigs | 508 |
| BNG (optical map) total length | 622.21 Mb |
| BNG (optical map) N50 | 1.62 Mb |

**Table S2. Statistics for the *BssSI* optical map.**

| # Molecules | 242,927 |
| --- | --- |
| Molecule N50 | 294.9 Kb |
| Molecule minimum length | 180 Kb |
| Molecule total length | 71.5 Gb |
| # BNG (optical map) contigs | 743 |
| BNG (optical map) total length | 577.76 Mb |
| BNG (optical map) N50 | 1.02 Mb |

**Table S3.** **Assembly statistics for the eight individual draft assemblies.**

|  | **CANU**^a^ | **CANU**^b^ | **ABruijn** | **FALCON** | **CANU**^c^ | **CANU**^d^ | **CANU**^e^ | **CANU**^f^ |
| --- | --- | --- | --- | --- | --- | --- | --- | --- |
| Input reads (Gbp) | 56.6 | 56.6 | N/A | N/A | 56.6 | 56.6 | 56.6 | 56.6 |
| Input reads corrected (Gbp) | 17.5 | 30.6 | 30.6 | 30.6 | 15.9 | 19.5 | 18.8 | 31.6 |
| Input reads corrected (fold) | 28.2x | 49.4x | 49.4x | 49.4x | 25.7x | 31.7x | 30.6x | 51.6x |
| N50 (bp) | 5,307,785 | 4,754,622 | 2,157,242 | 3,138,839 | 3,751,474 | 3,175,625 | 2,798,135 | 5,641,635 |
| L50 | 27 | 31 | 67 | 45 | 41 | 47 | 48 | 28 |
| NG50 (bp) | 3,966,144 | 3,849,144 | 1,510,173 | 2,084,336 | 2,758,179 | 2,303,516 | 2,264,855 | 4,070,253 |
| LG50 | 39 | 43 | 107 | 65 | 59 | 66 | 69 | 39 |
| total (bp) | 505,857,042 | 517,175,664 | 478,230,679 | 511,933,729 | 504,711,938 | 516,558,510 | 515,964,327 | 510,383,709 |
| contigs | 878 | 946 | 498 | 1,789 | 1,053 | 1,093 | 1,119 | 944 |
| contigs ≥100kbp | 200 | 277 | 399 | 376 | 281 | 338 | 313 | 265 |
| contigs  ≥ 1Mbp | 98 | 103 | 149 | 114 | 123 | 133 | 139 | 94 |
| contigs  ≥ 10Mp | 10 | 10 | 1 | 1 | 2 | 4 | 3 | 10 |
| longest contig (bp) | 18,457,871 | 18,479,951 | 12,614,652 | 10,554,495 | 14,090,735 | 14,493,815 | 13,236,031 | 17,184,516 |
| mapped SNPs | 49,804 | 49,863 | 49,710 | 49,587 | 49,847 | 49,830 | 49,823 | 49,863 |
|  |  |  |  |  |  |  |  |  |

**N50:** length for which the set of contigs of that length or longer accounts for at least half of the assembly size

**NG50:** length for which the set of contigs of that length or longer accounts for at least half of the ~620Mb genome

**L50:** minimum number of contigs accounting for at least half of the assembly

**LG50:** minimum number of contigs accounting for at least half of the ~620Mb genome

**Assembly parameters:** assembly tools were run with default parameters, except for the following CANU^a^ (corMhapSensitivity=low), CANU^b^ (corMhapSensitivity=low, corOutCoverage=100), ABruijn (k=19, cov=49), FALCON (default), CANU^c^ (corMhapSensitivity=high), CANU^d^ (corMhapSensitivity=high, corMaxEvidenceErate=0.15, corOutCoverage=100), CANU^e^ (corMhapSensitivity=normal, corMaxEvidenceErate=0.15, corOutCoverage=100), CANU^f^ (corMhapSensitivity=high corOutCoverage=100)

**Mapped SNPs:** number of SNP design sequenced mapped to each assembly with an e-score of 1e-50 or better (see text)

**Table S4. Characteristics of the 10 genetic maps used for pseudochromosome construction.** Cowpea chromosomes are numbered based on the new system (cross-reference shown in Table S5).

| **Genetic map** | **Characteristic** | **Vu01** | | **Vu02** | **Vu03** | **Vu04** | **Vu05** | **Vu06** | **Vu07** | **Vu08** | **Vu09** | **Vu10** | **Vu11** | **All** |
| --- | --- | --- | --- | --- | --- | --- | --- | --- | --- | --- | --- | --- | --- | --- |
| Tvu-14676 x IT84S-2246-4^5^ | Markers | | 1,207 | 953 | 2,765 | 1,066 | 1,563 | 1,326 | 1,447 | 1,618 | 1,250 | 774 | 691 | 14,660 |
|  | Bins | | 111 | 82 | 186 | 81 | 126 | 119 | 105 | 131 | 126 | 67 | 82 | 1,216 |
|  | cM | | 66.96 | 68.74 | 121.29 | 55.80 | 84.50 | 70.46 | 56.85 | 67.14 | 94.18 | 56.57 | 70.41 | 812.90 |
| Sanzi x Vita7^5^ | Markers | | 1,256 | 1,376 | 2,912 | 1,393 | 1,156 | 973 | 1,920 | 1,050 | 1,343 | 851 | 1,389 | 15,619 |
|  | Bins | | 115 | 77 | 215 | 103 | 92 | 85 | 157 | 112 | 116 | 83 | 110 | 1,265 |
|  | cM | | 77.59 | 50.97 | 156.40 | 84.95 | 82.07 | 70.13 | 101.01 | 85.03 | 100.46 | 66.37 | 80.51 | 955.51 |
| ZN016 x Zhijiang282^5^ | Markers | | 857 | 580 | 1,551 | 858 | 800 | 690 | 426 | 562 | 791 | 456 | 393 | 7,964 |
|  | Bins | | 55 | 57 | 123 | 49 | 70 | 60 | 49 | 56 | 83 | 43 | 52 | 697 |
|  | cM | | 71.49 | 65.27 | 124.01 | 45.09 | 91.70 | 62.88 | 54.48 | 75.47 | 94.07 | 56.64 | 62.28 | 803.38 |
| CB46 x IT93K-503-1^5^ | Markers | | 1,374 | 1,138 | 2,745 | 855 | 1,342 | 1,151 | 2,050 | 1,336 | 1,759 | 1,601 | 1,227 | 16,578 |
|  | Bins | | 88 | 92 | 179 | 66 | 109 | 74 | 109 | 91 | 116 | 83 | 76 | 1,083 |
|  | cM | | 73.09 | 67.87 | 132.85 | 56.00 | 94.54 | 57.64 | 84.46 | 67.26 | 85.50 | 62.87 | 59.77 | 841.84 |
| CB27 x IT82E-18^5^ | Markers | | 737 | 1,534 | 2,640 | 1,843 | 1,520 | 1,539 | 1,295 | 1,255 | 1,195 | 1,778 | 1,230 | 16,566 |
|  | Bins | | 37 | 68 | 159 | 93 | 100 | 62 | 100 | 105 | 109 | 69 | 75 | 977 |
|  | cM | | 59.07 | 46.54 | 132.64 | 82.06 | 95.31 | 60.01 | 78.47 | 90.07 | 86.69 | 59.76 | 68.30 | 858.91 |
| CB27 x IT97K-566-6 | Markers | | 1,577 | 1,205 | 3,174 | 1,230 | 796 | 1,576 | 1,212 | 803 | 1,721 | 1,735 | 1,255 | 16,284 |
|  | Bins | | 144 | 59 | 144 | 38 | 78 | 68 | 97 | 52 | 117 | 52 | 72 | 921 |
|  | cM | | 86.47 | 47.87 | 126.36 | 56.92 | 83.12 | 57.04 | 88.59 | 61.01 | 92.39 | 51.88 | 76.70 | 828 |
| 524B x IT84S-2049^27^ | Markers | | 738 | 981 | 2,483 | 1,234 | 942 | 1,425 | 1,257 | 1,311 | 1,626 | 961 | 1,244 | 14,202 |
|  | Bins | | 43 | 72 | 175 | 84 | 66 | 64 | 105 | 97 | 114 | 74 | 57 | 951 |
|  | cM | | 69.72 | 60.44 | 148.52 | 86.08 | 71.80 | 63.93 | 105.19 | 76.80 | 110.95 | 64.11 | 51.99 | 910 |
| CB46-Null x FN-2-9-04 | Markers | | 1,168 | 1,454 | 3,354 | 1,245 | 1,132 | 1,342 | 1,798 | 1,407 | 1,678 | 961 | 1,669 | 17,208 |
|  | Bins | | 126 | 106 | 250 | 102 | 82 | 108 | 147 | 121 | 151 | 91 | 108 | 1,392 |
|  | cM | | 80.94 | 74.72 | 150.83 | 92.78 | 60.02 | 65.30 | 110.66 | 81.61 | 107.51 | 76.27 | 85.25 | 986 |
| CB27 x UCR779 | Markers | | 1,565 | 1,475 | 3,684 | 1,481 | 1,103 | 1,556 | 1,792 | 1,635 | 1,519 | 772 | 1,632 | 18,214 |
|  | Bins | | 52 | 45 | 111 | 47 | 34 | 91 | 66 | 48 | 63 | 39 | 48 | 644 |
|  | cM | | 60.18 | 61.36 | 125.55 | 58.17 | 76.37 | 62.91 | 84.60 | 54.58 | 75.75 | 70.22 | 56.04 | 786 |
| IT99K-573-1-1 x TVNu-1158^9^ | Markers | | 1,742 | 1,368 | 2,780 | 117 | 1,740 | 1,676 | 1,968 | 1,607 | 1,774 | 1,416 | 1,551 | 17,739 |
|  | Bins | | 132 | 140 | 280 | 24 | 205 | 150 | 228 | 159 | 189 | 144 | 174 | 1,825 |
|  | cM | | 65.31 | 98.62 | 157.61 | 15.25 | 114.62 | 78.29 | 134.15 | 81.59 | 100.37 | 77.37 | 102.85 | 1,026 |

**Table S5. Cross-reference between old and new chromosome numbers for cowpea (Vu).** The total length of the syntenic matches (exact match >100 bp, alignment length > 1 kb) with the top two *P. vulgaris* (Pv) chromosomes is shown. Chromosomes that were inverted to meet the “short arm on top” convention are indicated in parenthesis. (*) Optimal solution.

| Old Vu Chr. |  | Pv Chr (kb) | Pv Chr (kb) | New Vu Chr. |
| --- | --- | --- | --- | --- |
| 1 |  | 8 (671.2) | **5** (485.1) | **5*** |
| 2 |  | **7** (1390.0) |  | **7** |
| 3 |  | **3** (1493.9) | 2 (899.8) | **3** |
| 4 |  | **1** (932.0) | 5 (245.0) | **1** |
| 5 |  | **8** (573.3) | 1 (309.0) | **8*** |
| 6 |  | **6** (996.8) |  | **6** (inverted) |
| 7 |  | **2** (736.9) | 3 (163.8) | **2** |
| 8 |  | **9** (1439.5) |  | **9** |
| 9 |  | **11** (751.8) |  | **11** (inverted) |
| 10 |  | **10** (593.9) |  | **10** (inverted) |
| 11 |  | **4** (564.1) |  | **4** |

**Table S6.** Annotated repeat abundances in cowpea. The major represented classes, super-families, and subgroups of transposable elements as determined by automated annotation and classified according to the scheme of Wicker et al. (2007), as well as other major repeat types are presented.

|  |  |  |  |  | % of genome | % of TE (bp) | Number | Number (%) | Sum (Mbp) | Average length (bp) |
| --- | --- | --- | --- | --- | --- | --- | --- | --- | --- | --- |
| All repeats | | |  |  | **49.53** |  |  |  |  |  |
| Mobile Element | | |  |  | **39.23** | **100.00** |  | **100.00** |  |  |
|  | **Class I: Retroelement (RXX)** | | |  | **33.17** | **84.55** | **241542** | **82.32** | **179.035** |  |
|  |  | LTR Retrotransposon (RLX) | | | 32.76 | 83.49 | 230558 | 80.92 | 170.146 |  |
|  |  |  | *Gypsy* (RLG) | | 18.32 | 46.70 | 97313 | 34.17 | 95.162 | 978 |
|  |  |  | *Copia* (RLC) | | 11.80 | 30.09 | 107705 | 37.80 | 61.317 | 569 |
|  |  |  | TRIMs | | 0.002 | 0.01 | 57 | 0.02 | 0.012 | 206 |
|  |  |  | unclassified LTR (RLX) | | 2.48 | 6.31 | 24637 | 8.65 | 12.860 | 522 |
|  |  | non-LTR Retrotransposon (RXX) | | | 0.41 | 1.06 | 3971 | 1.39 | 2.153 |  |
|  |  |  | LINE (RIX) | | 0.36 | 0.92 | 3571 | 1.25 | 1.883 | 527 |
|  |  |  | SINE (RSX) | | 0.05 | 0.13 | 400 | 0.14 | 0.269 | 673 |
|  | **Class II: DNA Transposon (DXX)** | | | | **6.06** | **15.45** | **50376** | **17.68** | **31.491** |  |
|  |  | DNA Transposon Superfamily (DTX) | | | 4.70 | 11.97 | 42902 | 15.06 | 24.402 |  |
|  |  |  | CACTA (DTC) | | 2.22 | 5.66 | 19538 | 6.86 | 11.538 | 591 |
|  |  |  | hAT (DTA) | | 1.38 | 3.51 | 15684 | 5.50 | 7.159 | 456 |
|  |  |  | MuDR (DTM) | | 0.94 | 2.40 | 6672 | 2.34 | 4.884 | 732 |
|  |  | MITE (DXX) | | | 0.05 | 0.11 | 218 | 0.08 | 0.233 | 1067 |
|  |  | Helitron (DHH) | |  | 1.30 | 3.31 | 7013 | 2.46 | 6.736 | 961 |
|  |  | unclassified DNA transposon (DXX) | | | 0.02 | 0.06 | 239 | 0.08 | 0.119 | 498 |
|  | **Class I/Class II ratio** | | |  | **7.23** |  | **5.57** |  |  |  |
|  | ***Gypsy/Copia* ratio** | |  |  | **1.55** |  | **0.90** |  |  |  |
| Other repeats | | | |  |  |  |  |  |  |  |
|  | Simple Sequence Repeats (SSRs) | | |  | 4.03 |  | 188510 |  | 20. 936 | 111 |
|  | Satellite repeats | | |  | 0.06 |  | 93 |  | 0.330 | 3551 |
|  | Ribosomal DNA (rDNA) | | |  | 0.49 |  | 505 |  | 2. 555 |  |
|  | Unknown low-complexity sequence | | |  | 5.68 |  | 76875 |  | 29.515 |  |

**Table S7. Centromere position prediction.** Start and end positions of centromeric regions based on BLAST of the 455-bp tandem repeat identified by Iwata-Otsubo et al. (2016).

| **Chromosome** | **Start (bp)** | **End (bp)** | **Range (bp)** |
| --- | --- | --- | --- |
| Vu01 | 14,698,036 | 16,525,496 | 1,827,460 |
| Vu02 | 10,238,236 | 14,020,258 | 3,782,022 |
| Vu03 | 30,476,981 | 31,470,261 | 993,280 |
| Vu04 | 19,069,641 | 21,130,843 | 2,061,202 |
| Vu05 | 25,704,431 | 33,885,354 | 8,180,923 |
| Vu06 | 9,156,830 | 9,235,637 | 78,807 |
| Vu07 | 16,587,031 | 16,604,960 | 17,929 |
| Vu08 | 14,914,119 | 15,164,402 | 250,283 |
| Vu09 | 20,802,610 | 22,685,597 | 1,882,987 |
| Vu10 | 18,917,563 | 19,028,450 | 110,887 |
| Vu11 | 17,283,961 | 18,283,861 | 999,900 |

**Table S8. Number and location of SNPs relative to annotated cowpea genes.**

|  | **1M list** | **iSelect** |
| --- | --- | --- |
| # SNPs | 957,710 | 51,128 |
| # SNPs in genes (%) | 336,285 (35%) | 31,708 (62%) |
| # SNPs in exons (%) | 138,892 (15%) | 16,898 (33%) |
| # SNPs in or within 1 kb from gene (%) | 460,709 (48%) | 38,286 (75%) |
| # SNPs in or within 2 kb from gene (%) | 540,773 (56%) | 39,856 (78%) |
| # SNPs in or within 10 kb from gene (%) | 792,318 (83%) | 45,648 (89%) |
| # unique genes with SNPs (%) | 23,266 (78%) | 17,444 (59%) |
| # unique genes containing or near SNPs (< 1 kb) (%) | 25,433 (85%) | 19,319 (65%) |
| # unique genes containing or near SNPs (< 2 kb) (%) | 26,130 (88%) | 19,818 (67%) |
| # unique genes containing or near SNPs (< 10 kb) (%) | 27,021 (91%) | 21,205 (71%) |

**Table S9. Comparative repeat abundance in *Vigna* species.** Repeats in each genome assembly were annotated by common methods and categorized into the major groups as in Table S6.

|  |  |  |  |  | *V. unguiculata*  (% genome) | *V. angularis*  (% genome) | *V. radiata*  (% genome) | Vu vs. Vr (Mbp) | Vu vs. Va (Mbp) | Va vs. Vr (Mbp) |
| --- | --- | --- | --- | --- | --- | --- | --- | --- | --- | --- |
| Genome assembly size (Mbp) | | | | | 519.44 | 467.30 | 463.64 | 55.798 | 52.135 | 3.663 |
| Mobile Element | | |  |  | **39.23** | **34.21** | **32.69** | **52.236** | **43.907** | **8.330** |
|  | **Class I: Retroelement (RXX)** | | |  | **33.17** | **31.93** | **30.13** | **32.599** | **23.113** | **9.486** |
|  |  | LTR Retrotransposon (RLX) | | | 32.76 | 31.68 | 29.83 | 31.836 | 22.103 | 9.733 |
|  |  |  | *Gypsy* (RLG) | | 18.32 | 15.12 | 14.02 | 30.155 | 24.525 | 5.631 |
|  |  |  | *Copia* (RLC) | | 11.80 | 11.68 | 11.49 | 8.061 | 6.723 | 1.338 |
|  |  |  | unclassified LTR (RLX) | | 2.48 | 4.85 | 4.22 | -6.692 | -9.801 | 3.108 |
|  |  | non-LTR Retrotransposon (RXX) | | | 0.41 | 0.12 | 0.12 | 1.577 | 1.584 | -0.008 |
|  |  |  | LINE (RIX) | | 0.36 | 0.11 | 0.12 | 1.343 | 1.362 | -0.019 |
|  |  |  | SINE (RSX) | | 0.05 | 0.01 | 0.01 | 0.234 | 0.223 | 0.011 |
|  | **Class II: DNA Transposon (DXX)** | | | | **6.06** | **2.29** | **2.16** | **21.457** | **20.794** | **0.664** |
|  |  | DNA Transposon Superfamily (DTX) | | | 4.70 | 2.19 | 2.10 | 14.675 | 14.161 | 0.514 |
|  |  |  | CACTA (DTC) | | 2.22 | 1.29 | 0.98 | 6.972 | 5.497 | 1.475 |
|  |  |  | hAT (DTA) | | 1.38 | 0.33 | 0.43 | 5.170 | 5.622 | -0.452 |
|  |  |  | MuDR (DTM) | | 0.94 | 0.50 | 0.60 | 2.100 | 2.555 | -0.455 |
|  |  | Helitron (DHH) | |  | 1.30 | 0.12 | 0.17 | 5.927 | 6.162 | -0.235 |
|  | **Class I/Class II ratio** | | |  | **7.2** | **13.9** | **13.9** |  |  |  |
|  | ***Gypsy*/*Copia* ratio** | |  |  | **1.55** | **1.22** | **1.29** |  |  |  |
| Other repeats | | | |  |  |  |  |  |  |  |
|  | Simple Sequence Repeats (SSRs) | | |  | 4.03 | 2.25 | 1.50 | 0.835 | -2.722 | 3.557 |

**Table S10.** **Primer sequences used to validate the Vu03 inversion.** PCR primer sequences designed to amplify the two breakpoints of the inversion using both the orientation of the reference and the opposite orientation.

| **Primer** | **Sequence** | **Tm (°C)** | **Amplicon size (bp)** | **Breakpoint target** | **Orientation** |
| --- | --- | --- | --- | --- | --- |
| BP1_Ref_Forward | CCTTGTCCCCCATTTTTCTT | 60.16 |  | 1 | Reference |
| BP1_Ref_Reverse | TGATGTGAAATTGTGATCCATGT | 60.11 | 822 | 1 | Reference |
| BP1_Opp_Forward | CCTTGTCCCCCATTTTTCTT | 60.16 |  | 1 | Opposite |
| BP1_Opp_Reverse | TTGAGCACCAAAGTGTCGAA | 60.43 | 796 | 1 | Opposite |
| BP2_Ref_Forward | AAAATGACCGGAATCATGAAC | 58.75 |  | 2 | Reference |
| BP2_Ref_Reverse | TTACCATTCGCAACGAAAAA | 59.19 | 389 | 2 | Reference |
| BP2_Opp_Forward | TGATGTGAAATTGTGATCCATGT | 60.11 |  | 2 | Opposite |
| BP2_Opp_Reverse | TTACCATTCGCAACGAAAAA | 59.19 | 822 | 2 | Opposite |

**Table S11.** **Data sources and references for genome assemblies and annotations used in the gene family analysis.**

| **Genus species** | **Abbreviation** | **Genotype** | **Assembly** | **Annotation** | **Citation** | **Source** | **Original filename** |
| --- | --- | --- | --- | --- | --- | --- | --- |
| *Vigna unguiculata* | vigun | IT97K-499-35 | 1 | 1 | This study | Phytozome | Vunguiculata_469_v1.1.protein_primaryTranscriptOnly.fa |
| *Vigna angularis* | vigan | Shumari | 1 | 1 | 74 | VigGS | Vangularis_v1.a1.protein.fasta |
| *Vigna radiata* | vigra | VC1973A | 6 | 1 | 39 | LegumeInfo | vigra.VC1973A.gnm6.ann1.M1Qs.protein.faa |
| *Phaseolus vulgaris* | phavu | G19833 | 2 | 1 | 23 | Phytozome | Pvulgaris_442_v2.1.protein.fa |
| *Cajanus cajan* | cajca | ICPL87119 | 1 | 1 | 24 | LegumeInfo | cajca.ICPL87119.gnm1.ann1.Y27M.protein_main.faa |
| *Glycine max* | glyma | Williams 82 | 2 | 1 | 30 | Phytozome | Gmax_275_Wm82.a2.v1.protein_primaryTranscriptOnly.fa |
| *Medicago truncatula* | medtr | A17_HM341 | 4 | 2 | 83 | LegumeInfo | medtr.A17_HM341.gnm4.ann2.G3ZY.pep.faa |
| *Cicer arietinum* | cicar | Frontier | 1 | 1 | 84 | LegumeInfo | cicar.CDCFrontier.gnm1.ann1.nRhs.gene.pep.faa |
| *Trifolium pratense* | tripr | MilvusB | 2 | 1 | 85 | LegumeInfo | tripr.MilvusB.gnm2.ann1.DFgp.protein_primaryTranscript.faa |
| *Lotus japonicus* | lotja | MG20 | 3 | 1 | 86 | LegumeInfo | lotja.MG20.gnm3.QPGB.protein.faa |
| *Lupinus angustifolius* | lupan | Tanjil | 1 | 1 | 87 | LegumeInfo | lupan.Tanjil.gnm1.ann1.nnV9.protein_all.faa |
| *Arachis duranensis* | aradu | V14167 | 1 | 1 | 88 | LegumeInfo | aradu.V14167.gnm1.ann1.cxSM.protein.faa |
| *Arachis ipaensis* | araip | K30076 | 1 | 1 | 88 | LegumeInfo | araip.K30076.gnm1.ann1.J37m.protein.faa |
| *Arachis hypogaea* | arahy | Tifrunner | 1 | 1 | Peanutbase | LegumeInfo | arahy.Tifrunner.gnm1.ann1.CCJH.protein_primaryTranscript.faa |
| *Arabidopsis thaliana* | arath | Col-0 | TAIR10 | 1 | TAIR | Phytozome | Athaliana_167_TAIR10.protein_primaryTranscriptOnly.fa |
| *Cucumis sativus* | cucsa | unknown | 1 | 1 | Phytozome | Phytozome | Csativus_122_v1.0.protein_primaryTranscriptOnly.fa |
| *Prunus persica* | prupe | Lovell | 2 | 2.1 | 89 | Phytozome | Ppersica_298_v2.1.protein_primaryTranscriptOnly.fa |
| *Solanum lycopersicum* | solly | Heinz_1706 | 2.5 | ITAG2.4 | 90 | Phytozome | Slycopersicum_390_ITAG2.4.protein_primaryTranscriptOnly.fa |
| *Vitis vinifera* | vitvi | PN40024 | Genoscope.12X | Genoscope.12X | 91 | Phytozome | Vvinifera_145_Genoscope.12X.protein_primaryTranscriptOnly.fa |
